# Photo-induced photoacoustic streaming and pineapple peel extract as final irrigant on extrusion bond strength of root filling material to canal wall

**DOI:** 10.12669/pjms.39.4.7370

**Published:** 2023

**Authors:** AbdulRahman Saeed AlGhamdi, Badi Alotaibi, Hanin Alsalhi, Roqayah Ibrahim Aljuailan, Ali Barakat

**Affiliations:** 1 AbdulRahman Saeed AlGhamdi, Department of Restorative and Prosthetic Dentistry, College of Dentistry, Dar Al Uloom University, Riyadh, Saudi Arabia; 2 Badi Alotaibi, Department of Conservative Dental Sciences, College of Dentistry, Qassim University, Al-Mulayda, Qassim, Saudi Arabia; 3 Hanin Alsalhi, Department of Conservative Dental Sciences, College of Dentistry, Qassim University, Al-Mulayda, Qassim, Saudi Arabia; 4 Roqayah Ibrahim Aljuailan, Department of Conservative Dental Sciences, College of Dentistry, Qassim University, Al-Mulayda, Qassim, Saudi Arabia; 5 Ali Barakat, Department of Restorative and Prosthetic Dentistry, College of Dentistry, Dar Al Uloom University, Riyadh, Saudi Arabia

**Keywords:** Photon-induced photoacoustic streaming, Sodium hypochlorite, Pineapple peel extract, Ethylenediaminetetraacetic acid

## Abstract

**Objective:**

Photon-induced photoacoustic streaming (PIPS) with pineapple peel extract (PPE) and ethylenediaminetetraacetic acid (EDTA) as a final endodontic irrigant on the push-out bond strength (PBS) of root filling material to conventional irrigation NaOCl with EDTA**.**

**Methods:**

An in vitro study at Dar Al Uloom University was conducted over three months. Root canal preparation was performed. Based on final irrigation, all the specimens were divided randomly into four groups (n=10) Group 1: 2.25% NaOCl+ 17% EDTA (control), Group-2: 2.25% NaOCl+PIPS + 6.25% PPE, Group-3: 2.25% NaOCl+PIPS + 17% EDTA, Group-4: 2.25% NaOCl + 6.25% PPE. Canals were obturated and sealed with AH Plus sealer. Root sectioning was performed at 1mm thickness and PBS testing was performed using the universal testing machine. The debonded samples were analyzed for failure mode. ANOVA compared the means and standard deviations (SD) of all investigated group. Assessment of multiple comparisons was performed using Tukey’s post hoc test.

**Results:**

The maximum PBS was demonstrated by the coronal section of Group-2 (2.25%NaOCl +PIPS+6.25%PPE) specimens (8.21±0.81MPa). The apical section of Group-1 (2.25% NaOCl+17% EDTA) specimens demonstrated minimum bond strength (2.80±0.18 MPa). The intergroup comparison revealed that Group-3 (2.25% NaOCl +PIPS +17% EDTA) and Group-2 demonstrated comparable outcomes (*p>*0.05). Group-4 (2.25% NaOCl +6.25% PPE) specimens established significantly lower values than Group-3 and Group-4 (*p<*0.05).

**Conclusion:**

Photon-induced photoacoustic streaming with pineapple peel extract and EDTA demonstrated better bond strength of root canal sealer and have the potential to be used as the final irrigant.

## INTRODUCTION

Due to the complex anatomy of the root canal system, it is impossible to achieve complete disinfection using instrumentation alone. Irrigation is an important counterpart to instrumentation because it removes the smear layer from the canal.^1^ Sealer penetration into the dentinal tubules is a desirable property because it would entomb residual debris and microorganisms and restrict them from getting nutrients. Moreover, deep endodontic sealer penetration is notably important because it increases the push-out bond strength (PBS) of the sealer resulting in better clinical success.^2^ Sodium Hypochloride (NaOCl), a gold standard root canal disinfectant has an extensive history of success in the field of dentistry.

It performs saponification and amino acid neutralization reactions that lead to the antimicrobial effect and tissue dissolution process.^3^ However, literature has revealed that it has weak action toward the removal of the inorganic part of the smear layer.^4^ A chelator Ethylenediaminetetraacetic acid (EDTA) has widespread acceptance in the field of dentistry as it reacts with the calcium ions of dentin and forms soluble calcium chelates.^5^ EDTA however, is unable to remove the organic part of the smear layer along with low antimicrobial activity and erosion of radicular dentin.^1^ A study by Pribadi et al., has also advocated that it does not perform complete disinfection of the canal at the apical section of the root.^6^

A herbal therapeutic, Pineapple peel extract (PPE) has gained attention as it contains active substances such as saponins, bromelain, polyphenol, and flavonoids. These active substances can lower the surface tension to remove organic and inorganic parts of the smear layer resulting in enhanced root canal cleanliness.^7^ Bromelain enzyme, on the other hand, performs antibacterial action by altering or damaging the wall structure of the bacterial protein.^7^ Recent work by Al AlKhatani et al., revealed that PPE improves the pushout bond strength (PBS) of glass fiber posts.^8^ However, its effect as a final root canal irrigant on the bond integrity of root filling material needs to be investigated.

Photon-induced photoacoustic streaming (PIPS) involves Er: YAG laser-activated root canal flushing which removes the smear layer from the canal thus increasing the bond integrity.^9^ Compared with other laser-activated irrigation (LAI), it is proven to be safe as it does not cause damage to the peri-radicular tissue. PIPS generates short microsecond pulse rates (50 μs) and low energy levels to achieve power peaks. These resulting photoacoustic shock waves generate 3D movement of the root canal disinfectant used.^10^,^11^

Based on current data available, it was identified that there is limited literature available that demonstrated the effect of newer root canal disinfection regimes (PPE and PIPS) on the PBS of root canal filling material. Therefore, it was hypothesized that there will be no significant difference in bond integrity of root filling material when the canal was disinfected using conventional final root canal irrigant 17% EDTA in comparison to that of contemporary disinfectant. The present study aimed to assess PIPS with PPE and EDTA and, NaOCl with PPE as a final endodontic irrigant on the push-out bond strength (PBS) of root filling material to conventional irrigation NaOCl with EDTA.

## METHODS

This in vitro study at Dar Al Uloom University was conducted over three months. Root canal preparation was performed. The study was approved by the Institutional Review Unit (IRU) (Ref.No. FR-368/m8).

### Preparation of PPE:

The one kg of 60 days old honey pineapple was peeled and dried out for about 24 hours. All the dried peel was submerged in 96% ethanol solution followed by placing them in the agitator (The Original Resinator OG®, USA). The agitated mix was protected using aluminum foil for 24 hours followed by filtering to produce filtrate one and residue. The residue obtained was therefore mixed with 96% ethanol solvent and placed in an agitator for two hours. The agitated mix was then filtered to generate filtrate-2. Instantly, filtrates-1 and 2 were combined and sealed off with the help of aluminum foil for 24 hours. The mixture of filtrate-1 and 2 was then strained to yield 1000 ml of solution. Using an evaporator the filtrate was concentrated at a temperature range of 50-60°C.^6^

### Specimen preparation:

Forty human premolar teeth having single roots extracted a-traumatically for orthodontic treatment were collected over a time duration of 30 days. Inclusion criteria consisted of teeth unveiling only a single straight canal established through X-rays and completely formed apices. However, teeth with any cracks, fractures, dilacerations, and calcification were excluded. Any attached calculus and debris were scaled off using ultrasonic scalers (Newtron Booster, Satelec- Acteon, Merignac, France). All the specimens before being used were then submerged in chloramine trihydrate solution (Merck, Mumbai, India) for 48 hours and preserved till the time of use. The present study was following CRIS (checklist for reporting in vitro study) guidelines.^12^

### Root canal preparation:

All the included teeth were rooted in acrylic mold and the coronal part of each specimen is sectioned till cementoenamel junction (CEJ) using a low-speed diamond disk (KG Sorensen, Ind. Com. Ltda.; Barueri, Sao Paolo, Brazil) maintaining the copious quantity of water irrigation. Root length was standardized at around 16 mm ±1 and working length was obtained by introducing the #15K file till the point it advanced across the root apex and then deducting 1 mm from that length. The root canal preparation was performed by using Pro Taper Next files (Dentsply Maillefer, Ballaigues, Switzerland) till the F3 finishing file. Between each file used and at the end of mechanochemical preparation canals were disinfected using a 2.25% NaOCl solution. Based on the final irrigation received, all the specimens were randomly divided into four groups. (n=10)^13^

### Group-1 (2.25% NaOCl + 17% EDTA) (control):

The canals were finally irrigated with five ml solution of 17% EDTA solution (Pulpdent, Oakland, USA) keeping it in the canal for one minute. This was followed by washing the canal with 5 ml of distilled water for 60 seconds.

### Group-2 (2.25% NaOCl+ PIPS + 6.25% PPE):

In this group, specimens were irrigated with the PIPS technique using 6.25% PPE as a final irrigant. In this technique canals were filled with 6.25% PPE solution and were irradiated with the help of Er: YAG laser (Syneron Medical LTD., Yokneam Israel), emitting light at a wavelength of 2,940 nm and adjusted at parameters at 0.4 W, 10 Hz, 40 mJ per pulse. The laser air flow was switched off and kept at the canal orifice during the irradiation process. The canals were then washed with 5 ml of distilled water for around 60 seconds.^14^,^15^

### Group-3 (2.25% NaOCl+ PIPS + 17% EDTA):

In this group, specimens were irrigated with the PIPS technique using EDTA as a final irrigant. In this technique, canals were filled with EDTA solution and were irradiated with the help of Er: YAG laser (Syneron Medical LTD., Yokneam Israel), emitting light at a wavelength of 2,940 nm and adjusted at parameters at 0.4 W, 10 Hz, 40 mJ per pulse. The laser air flow was switched off and kept at the canal orifice during the irradiation process. The canals were then washed with 5 ml of distilled water for around 60 seconds.

### Group-4 (2.25% NaOCl+6.25% PPE):

The canal was rinsed with 6.25% PPE solution keeping it for around 60 seconds in the canal followed by rinsing with distilled water. After the final irrigation of the canal with different disinfection regimes, canals were dried using absorbent paper points. Later the canals were obturated using gutta-percha (GP) using AH Plus sealer (Dentsply-Maillefer). All the obturated teeth were then submerged in normal saline for around seven days at a temperature of 37°C.

To assess the PBS of samples, root sectioning was performed in slices of one mm thickness starting one mm from the root apical area till the cementoenamel junction by placing a diamond blade disc in an Isomet machine (Isomet, Buehler, Lake Bluff, IL, USA) at a right angle to the root long axis under constant supply of water.

### Push-out testing:

The slices obtained were then applied with a compressive load with the help of a universal testing machine (BIET, Davangere, India). The load was applied at a crosshead speed of one mm/ minute with the help of stainless steel cylindrical plunger of different diameters i.e., (coronal third: one mm, middle third: 0.8 mm and apical thirds: and 0.3 mm) in corona-apical direction till the time GP filling was dislocated. The amount of force required to break the bond of sealer with the radicular dentin was calculated in Megapascals (MPa).

### Modes of failure and Statistical analysis:

The debonded samples were analyzed to assess the type of failure using a stereomicroscope (Olympus SZ61, Olympus Optical Co., Tokyo, Japan) at 40X magnification. The failure modes were classified according to the following criteria i.e., adhesive, cohesive, and admixed. Analysis of PBS and modes of failure will be accomplished using a statistical program for social science (IBM SPSS Statistics 21.0). Analysis of variance (ANOVA) compares the means and standard deviations (SD) of all investigational groups. Assessment of multiple comparisons was performed using Tukey’s post hoc test keeping the level of significance at *p*=0.05.

## RESULTS

PBS scores related to the experimental groups at all three-thirds of the canal (coronal, middle, and apical) are presented in Table-I. The maximum value of PBS was demonstrated by the coronal section of Group-2 (2.25% NaOCl+ PIPS + 6.25% PPE) specimens (8.21±0.81MPa). Nevertheless, the apical section of Group-1 specimens (2.25% NaOCl+ 17% EDTA) demonstrated and displayed the minimum values of bond integrity (2.80±0.18 MPa).

**Table-I:** Means and Standard deviations (SD) of extrusion bond strength (MPa) values among experimental groups at the cervical, middle, and apical levels of root.

Groups	Cervical	Middle	Apical
***Group-1:** 2.25% NaOCl+17% EDTA (Control)*	5.13±0.36 ^c, A^	4.15±0.42^c, A^	2.80±0.18^a, B^
***Group-2:** 2.25% NaOCl +PIPS+6.25% PPE*	8.21±0.81^a, A^	7.14±0.11^a,A^	5.71±0.12^b, B^
***Group-3:** 2.25% NaOCl**+** PIPS+17% EDTA*	8.17±0.43^a,A^	7.00±0.53^a,A^	5.33±0.26^b,B^
***Group-4:** 2.25% NaOCl + 6.25% PPE*	6.21±0.31^b, A^	5.31±0.55^b,A^	3.10±0.54^b, B^

Sodium hypochlorite (NaOCl); Ethylene diamine tetraacetic acid (EDTA); Pineapple peel extract (PPE); Photon-Induced Photoacoustic Streaming (PIPS); Different superscript lower-case alphabets denote statistically significant differences within the same column (p<0.05); Data with different upper-case alphabets denote significant differences within each row. (p<0.05).

The intergroup comparison analysis revealed that Group-3 (2.25% NaOCl+PIPS+17% EDTA), coronal (8.17±0.43 MPa), middle (7.00±0.53 MPa), and apical (5.33±0.26 MPa) and Group-2 (PIPS + 6.25% PPE), coronal (8.21±0.81 MPa), middle (7.14±0.11 MPa) and apical (5.71±0.12 MPa) specimens at all three-thirds of the root section demonstrated the comparable outcomes of PBS (*p>*0.05). Conversely, Group-4 (2.25% NaOCl+6.25% PPE) specimens established significantly lower values at all three thirds, coronal (6.21±0.31 MPa), middle (5.31±0.55 MPa), and apical (3.10±0.54 MPa) than Group-2 and Group-3. (*p<* 0.05) Moreover, it was identified that in Group-1 specimens all three sections of root displayed significantly lowest values of bond integrity at coronal (5.13±0.36 MPa), middle (4.15±0.42 MPa), and apical (2.80±0.18 MPa) thirds from the other tested groups.

**Fig.1:**
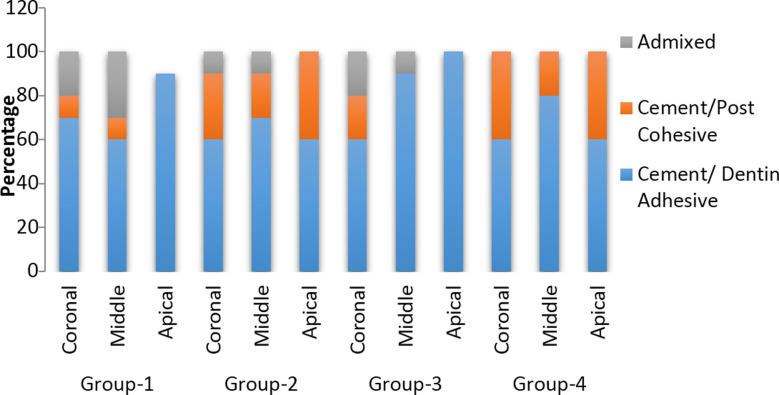
Percentage of modes of failure in each group.

Intragroup comparison displayed that in all the experimental groups tested, coronal and middle sections of the root demonstrated comparable outcomes of bond integrity (*p>*0.05) However, the bond strength decreases significantly at the apical section of the root (*p<* 0.05). The modes of failure percentage of all the tested groups are exhibited in Fig-1. It was identified that all the groups displayed the adhesive type of failure as the most common mode of failure followed by cohesive and admixed failure patterns.

## DISCUSSION

The existing study was based on the hypothesis that there will be no significant difference in bond integrity of root filling material when the canal was disinfected using conventional final root irrigant 17% EDTA in comparison to that of contemporary disinfectant. The postulated hypothesis was rejected as all the modern final disinfection regimes displayed higher values of PBS than the control. The push-out test was used to assess the sealing capacity of root canal obturation materials. It is also considered a valuable tool to evaluate bond sores of different root canal sealers bonded to radicular dentin and help to classify materials following their adhesive.^16^,^17^

The results of the present study indicated that 2.25%NaOCl+PIPS+6.25%PPE treated specimens demonstrated the highest values of PBS as compared to the other groups tested. The outcome can be explained by synergistic action displayed by PIPS and PPE. Pribadi et al., and Alkhudhairy et al., displayed in their study that PPE performs better canal disinfection and smear layer removal than conventional EDTA solution.^6^,^18^ The high smear layer removal effect of 6.25% PPE is due to the presence of active constituents such as saponins, bromelain, polyphenol, and flavonoids. All work in harmony to eliminate the smear layer.^19^

PIPS technique on the other hand also possesses the ability to expose organic collagen fibers which results in the generation of an uneven and irregular root surface that permits the adhesive resin sealer to penetrate the dentin surface resulting in the enhancement of mechanical interlocking thus improving the bond strength of AH plus sealer.^9^ Group-3 specimens treated with 2.25% NaOCl+PIPS +17% EDTA also exhibited comparable outcomes of bond integrity to that of 2.25% NaOCl+PIPS + 6.25% PPE treated samples. This is in line with the findings of the study conducted by Wen at el.,^20^ They identify the effect of PIPS using MTAD solution and suggested that the combination technique used is effective in getting rid of the smear layer.

Moreover, results also exhibited that 2.25% NaOCl+PPE when used alone as a final root canal irrigant gives lower PBS than the PIPS-treated groups. However, it demonstrated significantly better bond strength than the group treated with 2.25% NaOCl+EDTA. The higher bond strength achieved by PPE than EDTA-treated specimens can be explained by the presence of a potentially active substance which improves smear layer removal and promotes bond strength.^21^,^22^ It can also be stated that EDTA and PPE in combination with PIPS as a final irrigation protocol displayed better bond integrity than PPE and EDTA alone.

This indicated that the PIPS technique enhances the smear layer removing the ability of PPE and EDTA. A study by Wan et al. and Yameen et al., explained that Er: YAG laser possesses the highest absorption capacity in water and also displays an extraordinary affinity for hydroxyapatite crystals.^22^,^23^ Regarding modes of failure, it was identified that all the experimental groups mostly displayed the adhesive type of failure. The prevalence of adhesive failures at the sealer dentin interface is in line with the findings of previous literature and can be explained by the fact that complete drying of the canal is not possible and the moisture present may be the reason for the weak bond at the sealer dentin interface.^24^,^25^

### Limitations of the study:

The present research displayed some inherent limitations. Primarily, the in vitro nature of the study restricts the generalization of the findings as multiple factors influence restoration in the oral environment and may affect the outcome in a clinical setting. Similarly, the variation present in each sample related to the dentinal structure might have affected the results. Different concentrations of PPE and their effect on the PBS of the canal sealer need further investigation and probing. Electron microscopy and energy dispersive spectrometry should have been performed to assess the topography of root canal dentin.

## CONCLUSION

Photon-induced photoacoustic streaming with pineapple peel extract and EDTA demonstrated the better bond strength of root canal sealer and have the potential to be used as the final irrigant.

### Authors Contribution:

**ASA,BA:** Data collection, study design, manuscript writing, final manuscript approval.

**ASA,HA:** Data collection, study design, manuscript drafting, data analysis, manuscript approval.

**BA, RIA:** Data collection, manuscript approval, and data interpretation.

**RIA,HA:** Data collection, writing, revision, editing, and final manuscript approval.

**AB, RIA**: Rewrriting, revision, editing, and final manuscript approval

All authors are responsible and accountable for the accuracy and integrity of the work.

## References

[1] Wachlarowicz AJ, Joyce AP, Roberts S, Pashley DH (2007). Effect of Endodontic Irrigants on the Shear Bond Strength of Epiphany Sealer to Dentin. J Endod.

[2] Ok E, Ertas H, Saygili G, Gok T (2014). Effect of photo-activated disinfection on bond strength of three different root canal sealers. Eur J Dent.

[3] Salles MM, Badaro MM, Arruda CN, Leite VM, Silva CH, Watanabe E (2015). Antimicrobial activity of complete denture cleanser solutions based on sodium hypochlorite and Ricinus communis –A randomized clinical study. J Appl Oral Sci.

[4] Estrela C, Estrela CRA, Barbin EL, Spano JCE, Marchesan MA, Pecora JD (2002). Mechanism of action of sodium hypochlorite. Braz Dent J.

[5] Guo X, Miao H, Li L, Zhang S, Zhou D, Lu Y (2014). Efficacy of four different irrigation techniques combined with 60°C 3% sodium hypochlorite and 17% EDTA in smear layer removal. BMC Oral Health.

[6] Pribadi N, Samadi K, Astuti MNK, Kurniawan HJ, Tandadjaja AK, Hadi RP (2019). The differences in root canal smear layer removal between 6,25% pineapple (Ananas comocus L. Merr.) peel extract and 17% Ethylene diamine tetra-acetic acid. Dent J (Majalah Kedokt Gigi).

[7] Praveen NC, Rajesh A, Madan M, Chaurasia VR, Hiremath N V, Sharma AM (2014). In vitro Evaluation of Antibacterial Efficacy of Pineapple Extract (Bromelain) on Periodontal Pathogens. J Int oral Heal JIOH.

[8] Alkahtany MF (2022). Extrusion bond strength of glass fiber post to radicular dentin after final irrigation using MTAD, EDTA, Pineapple peel extract, and Riboflavin. Photodiagnosis Photodyn Ther.

[9] Wan S, Tan Y, Xie J, Huang X, Guo L (2020). The effect of a root-dentin pretreatment technique combining PIPS with MTAD aiming to improve the bond strength of glass fiber post. Microsc Res Tech.

[10] Vohra F, Bukhari IA, Sheikh SA, Naseem M, Hussain M (2020). Photodynamic activation of irrigation (using different laser prototypes) on push out bond strength of fiber posts. Photodiagnosis Photodyn Ther.

[11] Alkhudhairy F, Naseem M, Ahmad ZH, Alnooh AN, Vohra F (2019). Efficacy of phototherapy with different conventional surface treatments on adhesive quality of lithium disilicate ceramics. Photodiagnosis Photodyn Ther.

[12] Al Deeb L, Bin-Shuwaish MS, Abrar E, Naseem M, Al-Hamdan RS, Maawadh AM (2020). Efficacy of chlorhexidine, Er Cr YSGG laser and photodynamic therapy on the adhesive bond integrity of caries affected dentin. An in-vitro study. Photodiagnosis Photodyn Ther.

[13] Alrahlah A, Naseem M, Tanveer SA, Abrar E, Charania A, AlRifaiy MQ (2020). Influence of disinfection of caries effected dentin with different concentration of silver diamine fluoride, curcumin and Er, Cr:YSGG on adhesive bond strength to resin composite. Photodiagnosis Photodyn Ther.

[14] Alkhudhairy F, Vohra F, Naseem M, Ahmad ZH (2019). Adhesive bond integrity of dentin conditioned by photobiomodulation and bonded to bioactive restorative material. Photodiagnosis Photodyn Ther.

[15] Vohra F, Bukhari IA, Sheikh SA, Naseem M, Hussain M (2020). Photodynamic activation of irrigation (using different laser prototypes) on push out bond strength of fiber posts. Photodiagnosis Photodyn Ther.

[16] Al Ahdal K, Al Deeb L, Al-Hamdan RS, Bin-Shuwaish MS, Al Deeb M, Maawadh AM (2020). Influence of different photosensitizers on push-out bond strength of fiber post to radicular dentin. Photodiagnosis Photodyn Ther.

[17] Alkhudhairy FI, Alkheraif A, Bin-Shuwaish MS, Al-Johany S, Naseem M, Vohra F (2020). Effect of Er,Cr:YSGG Laser and Ascorbic Acid on the Bond Strength and Microleakage of Bleached Enamel Surface. Photodiagnosis Photodyn Ther.

[18] Alkhudhairy F, Naseem M, Bin-Shuwaish M, Vohra F (2018). Efficacy of Er Cr:YSGG laser therapy at different frequency and power levels on bond integrity of composite to bleached enamel. Photodiagnosis Photodyn Ther.

[19] Rahmi H, Widayanti A, Hanif A Utilization of Bromelain Enzyme from Pineapple Peel Waste on Mouthwash Formula Against Streptococcus mutans. IOP Conference Series:Earth and Environmental Science.

[20] Wen C, Kong Y, Zhao J, Li Y, Shen Y, Yang X (2021). Effectiveness of photon-initiated photoacoustic streaming in root canal models with different diameters or tapers. BMC Oral Health.

[21] Montero-Miralles P, Torres-Lagares D, Segura-Egea JJ, Serrera-Figallo MA, Gutierrez-Perez JL, Castillo-Dali G (2018). Comparative study of debris and smear layer removal with EDTA and Er,Cr:YSGG laser. J Clin Exp Dent.

[22] Wan S, Tan Y, Xie J, Huang X, Guo L (2020). The effect of a root-dentin pretreatment technique combining PIPS with MTAD aiming to improve the bond strength of glass fiber post. Microsc Res Tech.

[23] Yumeen S, Khan T (2022). Laser Erbium-Yag Resurfacing. StatPearls Publishing.

[24] Ehsani S, Bolhari B, Etemadi A, Ghorbanzadeh A, Sabet Y, Nosrat A (2013). The effect of Er,Cr:YSGG laser irradiation on the push-out bond strength of realseal self-etch sealer. Photomed Laser Surg.

[25] Oliveira KV, Silva BM, Leonardi DP, Crozeta BM, Sousa-Neto MD, Baratto-Filho F (2017). Effectiveness of different final irrigation techniques and placement of endodontic sealer into dentinal tubules. Braz Oral Res.

